# Eye Movement Control

**DOI:** 10.1155/2014/262541

**Published:** 2014-07-21

**Authors:** Stefanie I. Becker, Gernot Horstmann, Arvid Herwig

**Affiliations:** ^1^School of Psychology, University of Queensland, Brisbane, QLD 4072, Australia; ^2^Department of Psychology, Bielefeld University, 3361 Bielefeld, Germany

is well-known that eye movements are central to visual perception [1]. Visual acuity decreases dramatically in the periphery of vision, and precise eye movements to specific locations are vital to foveate objects of interest and identify them with high accuracy [1–4]. Given the importance of eye movements for visual perception, there has been a surge of interest in the topic, with numerous studies being conducted to clarify the variables that determine our eye movements (for a historical review see [5]).

In fact, Google Scholar shows that eye movements are discussed in over a million publications, and a Web of Science search reveals 17,000 publications with eye movement in the title or abstract. As shown in Figure 1, the number of publications with eye movement in the title or abstract has also steadily increased over years, culminating in about 200 papers published in 2013.

Despite the surge of interest in eye movements, many questions remain unresolved. This is also reflected in this special issue on eye movement control. First, there are a variety of different eye movements [2, 4]. Among the most widely known eye movements are the fast, ballistic saccades (including superfast express saccades) (e.g., B. de Gelder et al., this issue), smooth-pursuit eye movements (J. N. van der Geest et al., this issue), and vergence eye movements (e.g., P. M. Grove et al., this issue) required to fixate objects at different depths. Less well-known and yet intensely researched are microsaccades, tremor, slow drift, and vestibuloocular and optokinetic eye movements that stabilize gaze during motions of the head and motions of large regions of the image on the retina [2, 4].

Secondly and more importantly for the current special issue, eye movements are also controlled by a variety of different factors [1, 4]. Apart from being subject to diverse muscular and ocular constraints, successful voluntary control over eye movements critically depends on the quality of the visual input, which in turn depends on a variety of internal and external factors [1, 6, 7]. The contributions to the present special issue clarify key elements of both internal and external factors in eye movement control (G. W. Alpers et al., U. Ansorge et al., B. de Gelder et al., P. M. Grove et al., D. R. Hardwick et al., W. E. Huddlestone et al., J. Kassubek et al., A. Khan et al., A. Piras et al., N. D. Smith et al., J. N. van der Geest et al., and D. Venini et al., this issue).

In the present contributions, eye movements have also been used to provide new insights into ocular and neurological disorders (J. Kassubek et al., N. D. Smith et al., this issue) and shed new light on the relationship between covert attention and eye movements (e.g., G. W. Alpers et al., U. Ansorge et al., D. R. Hardwick et al., and A. Khan et al., this issue; see also [6–9]). Together, the papers in this special issue provide a timely update on eye movement control that reflects current hot topics in the field, spanning the range from cognitive science over applied psychology to clinical psychology and neuroscience.

*Stefanie I. Becker* *Stefanie I. Becker* *Gernot Horstmann* *Gernot Horstmann* *Arvid Herwig* *Arvid Herwig*

## Figures and Tables

**Figure 1:**
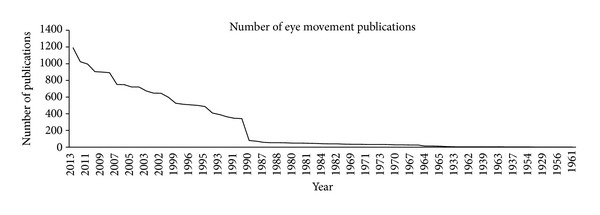
The number of publications with the “eye movement” in the title or abstract, according to a Web of Science search 2014.
